# Investigating carrier localization and transfer in InGaN/GaN quantum wells with V-pits using near-field scanning optical microscopy and correlation analysis

**DOI:** 10.1038/srep42221

**Published:** 2017-02-13

**Authors:** MinKwan Kim, Sunghan Choi, Joo-Hyung Lee, ChungHyun Park, Tae-Hoon Chung, Jong Hyeob Baek, Yong-Hoon Cho

**Affiliations:** 1 Graduate School of Nanoscience and Technology, Korea Advanced Institute of Science and Technology, Daejeon 34141, Republic of Korea; 2 Department of Physics, Korea Advanced Institute of Science and Technology, Daejeon 34141, Republic of Korea; 3 KI for the NanoCentury, Korea Advanced Institute of Science and Technology, Daejeon 34141, Republic of Korea; 4 LED Research and Business Division, Korea Photonics Technology Institute, Gwangju 61007, Republic of Korea

## Abstract

The V-pits and potential fluctuations in InGaN/GaN multiple quantum wells (MQWs) are key factors for understanding the performance of InGaN/GaN-based light-emitting diodes (LEDs). However, photoluminescence (PL) measurements using conventional optical microscopy only provide ensemble information due to the spatial resolution limit, known as the diffraction barrier, which hinders the analysis of dislocations and potential fluctuations. Here, in order to investigate the influence of the V-pits and potential fluctuations on local optical properties, we performed nanoscopic luminescence mapping for standard and V-pit InGaN/GaN MQWs samples with different sized V-pits using near-field scanning optical microscopy (NSOM) with illumination mode (I-mode) at various laser excitation powers. From the nanoscopic PL mapping data, we could clearly observe luminescence features associated with dislocations and potential fluctuations in the InGaN/GaN MQWs. We also employed correlation analysis to quantitatively analyze the nanoscopic PL mapping data for the different MQWs samples. Based on the results of NSOM PL with I-mode and correlation analysis, we could demonstrate that carrier transfer in the MQWs sample with large sized V-pits is suppressed by deeper potential fluctuations and higher energy barriers compared to the standard sample.

InGaN/GaN based light-emitting diodes (LEDs) have attracted tremendous attention for a wide range of applications, including displays and general illumination^1^,^2^,^3^. Among other advantages, they offer low energy consumption and an optical band gap that can be tuned from the visible to the ultraviolet spectral range by adjusting the indium composition^4^,^5^,^6^,^7^,^8^,^9^. Despite their popularity, many questions still remain about the factors that influence their emission efficiency. For example, InGaN/GaN LEDs exhibit high emission efficiency despite having high density ( > 10^8^ cm^−2^) of threading dislocation (TD)^10^,^11^,^12^. Such dislocations, which are induced by lattice mismatch between GaN and structurally dissimilar substrates, usually act as one of non-radiative recombination centers and carrier leakage channels, leading to low emission properties^13^,^14^,^15^,^16^,^17^,^18^. It has recently been proposed that the high efficiency of InGaN/GaN LEDs is caused by the formation of thin quantum wells (QWs) on the inclined facets around V-pits generated at the end of TDs, which prevents non-radiative recombination processes at the TDs^19^,^20^,^21^,^22^,^23^,^24^,^25^,^26^,^27^,^28^. Because these high energy QWs form around V-pits, some research have proposed methods for growing large sized V-pits to further enhance the efficiency of InGaN/GaN LEDs^29^,^30^,^31^,^32^. Meanwhile, it has been known that potential fluctuations owing to indium composition inhomogeneity and thickness variations can cause carrier localization and hence hinder non-radiative recombination processes in the InGaN/GaN multiple QWs (MQWs)^33^,^34^,^35^. According to recent studies, the potential fluctuations also seem to be affected by the formation of the V-pits at TDs due to strain relaxation around the V-pits^36^,^37^,^38^. Therefore, to improve the efficiency of InGaN/GaN LEDs, it is important to understand how the V-pits and potential fluctuations influence the optical properties of the LEDs in detail. However, optical investigation of V-pits and potential fluctuations using conventional microscopic photoluminescence (PL) techniques is difficult due to their limited spatial resolution, as V-pits and potential fluctuations occur in a sub-diffraction limit scale.

In this study, a nanoscopic PL technique using near-field scanning optical microscopy (NSOM PL) was employed to investigate the influence of V-pits and potential fluctuations on local optical properties. NSOM overcomes the spatial resolution limit by detecting the near field, including high spatial frequency components which exponentially decay near the sample surface, using a metal-coated tip with aperture and a scanning probe microscopy technique. NSOM PL measurements have been used for analysis of local fluctuation properties^39^,^40^,^41^,^42^,^43^,^44^,^45^ and for investigation of indium composition variations and relation between potential barriers around dislocations^46^ in InGaN QWs due to its ability to obtain mappings with sub-diffraction resolution. In our work, we used NSOM PL with the illumination mode (I-mode) and a systematic correlation analysis. In order to verify the influence of V-pits and potential fluctuations, two types of samples having different sized V-pits were used. By comparing the two samples, we observed influence of the V-pits and potential fluctuations, using power dependent NSOM PL mapping results. Correlation analysis, a statistical method, was employed as it can provide quantitative values from nanoscopically analyzed information^39^,^43^,^44^,^45^,^46^. Using correlation analysis, the two samples were found to exhibit quantitatively different correlation tendencies in terms of local carrier density, based on the low and high power NSOM PL results of each sample. Furthermore, we were able to estimate how carrier transfer can be affected by the V-pits and potential fluctuations in the InGaN/GaN MQWs of each sample, based on the results of NSOM PL and correlation analysis.

## Experiment

**Figure 1:**
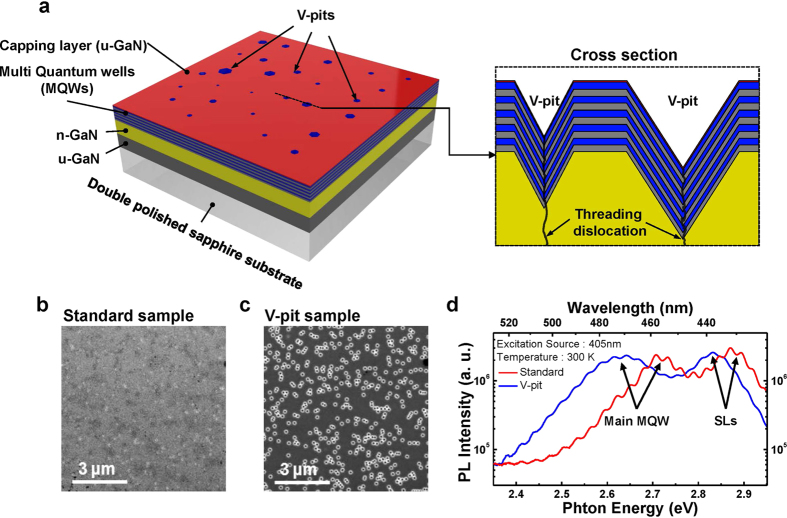
Schematic of sample structure and PL spectrum with SEM image. (**a**) Schematic of an InGaN/GaN blue MQWs sample grown on a double polished sapphire substrate. Two types of samples with different sized V-pit, called the standard and V-pit samples, were used. Schematic of cross section of a V-pit structure is provided in the inset image. (**b,c**) SEM images of the standard sample and the V-pit sample. As the SEM images show, the V-pit sample has larger sized V-pits compared to that of the standard sample. (**d**) PL spectra of the standard and the V-pit samples are measured at 300 K.

Two types of blue InGaN/GaN MQWs with different sized V-pits, hereafter designated standard and V-pit samples, were used to investigate the influence of V-pits and potential fluctuations. The epitaxial structures were comprised of 4-μm-thick n-doped GaN (n-GaN), 3.5-μm-thick un-doped GaN (u-GaN), and 90-nm-thick MQW layers, including five pairs of In_x_Ga_1-x_N/GaN main MQWs with x ~0.2 and four pairs of In_y_Ga_1-y_N/GaN superlattices (SLs) with y ~0.15 as a strain reducing layer, which were grown on sapphire substrates by metal-organic chemical vapor deposition, as shown in Fig. 1a. We note that the optical and electrical properties of the V-pit sample were found to be much improved compared to the standard sample. Detailed performance data on the V-pit LEDs will be published elsewhere (Chung *et al*., manuscript in preparation). For our NSOM PL experiments, double polished sapphire substrate was utilized as the substrate in the growth process to prevent emission signal scattering. A 1.5 nm un-doped GaN capping layer was grown as the top layer to protect the MQWs. A sample schematic of cross-section of a V-pit region is provided in the inset of Fig. 1a. Scanning electron microscopy (SEM) images of each sample are shown in Fig. 1b and c, respectively. As shown in the SEM images, the V-pit sample has large sized V-pits ( < 250 nm) at the TDs compared to the standard sample ( < 100 nm). The emission spectra of the standard and V-pit samples were measured by conventional macroscopic PL using a 405 nm excitation source at room temperature, as shown in Fig. 1d. Two peaks are found in the emission spectrum of each sample: one peak, observed in the high energy region, comes from the InGaN/GaN SLs, while the other peak observed in the low energy region comes from the main InGaN/GaN MQWs. The MQWs peak of the V-pit sample shows a red-shift compared to the standard sample. This red-shift of the spectrum suggests that the InGaN/GaN MQWs with the large sized V-pits has a longer emission wavelength compared to the standard InGaN/GaN MQWs. Based on this result, the red-shift of the far-field spectrum in V-pit sample can be attributed to strain relaxation and/or indium segregation caused by large sized V-pits. According to some research, V-pits were formed at dislocations to relax strain and indium can be easily incorporated at the outside region around the V-pits due to strain relaxation^21^,^37^,^47^. On the other hand, some research reported that similar V-pit feature could be made by selective etching at dislocations and strain relaxation becomes larger with increasing etching time^48^,^49^. Thus, the large sized V-pits with high density seem to cause a strain relaxation on the sample surface, leading to more indium incorporation during the growth due to lattice pulling effect^50^. Similar red-shift spectra of InGaN/GaN QWs caused by the large sized V-pits were also reported^25^.

**Figure 2:**
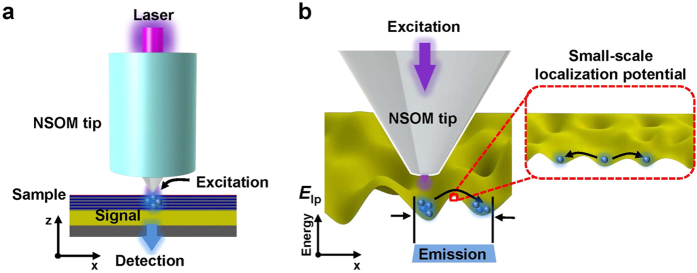
Schematics of the I-mode NSOM PL and emission region depending on localization potential. (**a**) Schematic of the I-mode NSOM PL. In operation, the I-mode NSOM PL excitation laser illuminates through the NSOM tip, and the luminescence of the sample is collected by the objective lens. (**b**) Schematic of the ‘emission region’ of the I-mode NSOM PL depending on localization potential at local potential fluctuation *E*_lp_.

To verify the influence of the V-pits and potential fluctuations in the nanoscale, each sample was measured using a tuning fork based NSOM PL with I-mode, at a laser power in the range of 100 to 1140 μW at room temperature. In the NSOM PL experiment, a 405 nm laser diode (L405P150, Thorlab, USA) was used as the excitation source and an aluminum coated optical fiber with a 100 nm aperture was used as the NSOM tip (MF001, NT-MDT, Russia). The photo excited carrier densities, which are evaluated by the excitation power carrier density (100 to 1140 μW), probe throughput, aperture diameter, and InGaN absorption coefficient, were between 3 × 10^9^ to 5 × 10^11^ cm^−2^. A schematic of the I-mode NSOM PL is shown in Fig. 2a. In operation, the excitation laser illuminated the sample through the NSOM tip (local excitation) and PL emission signals from the sample were collected through an objective lens (global detection). Because of the local excitation and the global detection in the I-mode, ‘emission region’ created by the local excitation is strongly affected by the potential fluctuations and energy barriers of the V-pits in the sample as shown in Fig. 2b. For example, if carriers are locally created by local excitation from the NSOM tip in sample with deep potential fluctuations, or high potential barriers, the carriers cannot diffuse far away, and then the NSOM PL will observe a small size of ‘emission region’. On the other hand, if carriers are locally created through local excitation by the NSOM tip in sample with shallow potential fluctuations, or low potential barriers, the NSOM PL will observe a large size of ‘emission region’. However, according to the recent results about local potential fluctuations in InGaN QWs^42^,^51^,^52^,^53^,^54^, potential fluctuations can be divided into large-scale localization potential (hundreds of nanometers) and small-scale localization potential (a few tens of nanometers). It was suggested that small-scale localization potential strongly affects the carrier transport and recombination compared to large-scale localization potential. By considering effect of small-scale localization potential, the schematics of ‘emission region’ of the I-mode NSOM PL are provided in inset of Fig. 2b. Whereas carriers in sample with deep small-scale localization potential are not able to diffuse far away due to suppressed transport caused by deep potentials, carriers in sample with shallow small-scale localization potential are able to widely diffuse. Finally, the PL emission signals collected from the ‘emission region’ were spectrally resolved by a monochromator (SP-2300i, Princeton Instruments, USA) and detected by an electron multiplying charge coupled detector (ProEM, Princeton Instruments, USA; see further details of the NSOM PL set-up in Methods), and then reconstructed point by point as an NSOM PL mapping image. In the power dependent PL case, the change in carrier density affects carriers escaping from the potential fluctuations in the QWs under the same temperature condition^43^. Using the ‘emission region’ schematic of the I-mode NSOM PL and the power dependent PL, we nanoscopically verified the influence of the V-pits and potential fluctuations in each sample.

## Results and Discussion

**Figure 3:**
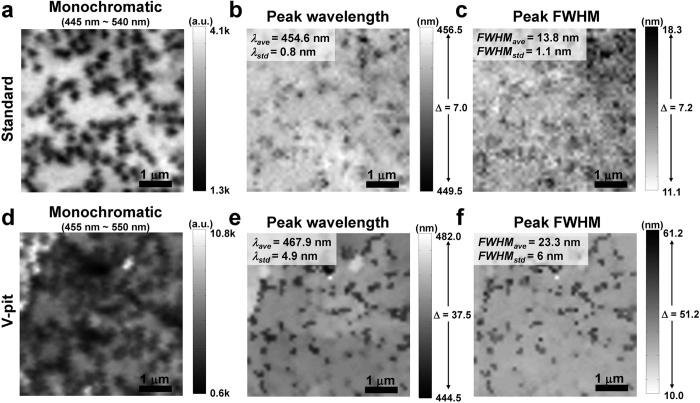
NSOM PL mapping images obtained from standard and V-pit samples at 100 μW laser power. Monochromatic NSOM PL images of the standard and V-pit samples, only obtained from main MQWs wavelength regime, are shown in (**a**) and (**d**), respectively. Using double-peak Gaussian fitting, mappings of peak wavelength and FWHM were also obtained. (**b,c**) Peak wavelength and FWHM mapping images of the standard sample. (**e,f**) Peak wavelength and FWHM mapping images of the V-pit sample. Inset values (*λ*_*ave*_*, λ*_*std*_*, FWHM*_*ave*_*, FWHM*_*std*_) show average and standard deviation values of the peak wavelength and FWHM. The values of Δ in the color bars of the peak wavelength and peak FWHM images indicate the differences between the maximum and minimum wavelengths and FWHM, respectively. In the monochromatic and peak FWHM images, we used white and black in the scale bars of the maximum intensity value, respectively, for better comparison with each other.

Monochromatic NSOM PL mapping images of the standard and V-pit samples obtained only from the main MQWs wavelength regime with 100 μW laser power are shown in Fig. 3a and d, respectively. The NSOM PL mapping size was 5 μm × 5 μm for each sample. Non-radiative recombination centers (NRCs) related to dislocations are clearly observed as the low intensity areas in each monochromatic NSOM PL image. Large spatial intensity fluctuations were also observed in the V-pit sample, as shown in Fig. 3d. The emission spectrum of each sample consists of two peaks which come from the main MQWs and the InGaN/GaN SLs. Each near-field double peak spectrum was fitted using double Gaussian fitting in order to completely separate and obtain spectrum information of main MQWs without emission of InGaN/GaN SLs. The peak wavelength and FWHM mappings of each sample were obtained, and are shown in Fig. 3b,c and e,f, respectively. By comparing mapping images, we determined that there were obvious correlations between the monochromatic and peak wavelength mappings, and between the monochromatic and peak FWHM mappings. For the monochromatic and peak wavelength mappings, the stronger intensity regions exhibited a longer peak wavelength tendency. Moreover, based on the comparison between the monochromatic and peak FWHM mappings, it was found that the stronger intensity regions exhibited a narrower FWHM tendency. These correlations not only suggest that radiative carrier recombination processes occur more often in the In-rich or thick QW areas, but also that the low intensity regions are related to NRCs or the spatial variations of the radiative recombination rate which may occur due to separate localization of electrons and holes at different regions^51^. However, the V-pit sample obviously shows stronger fluctuations in intensity, wavelength and FWHM compared to the standard sample. These fluctuations are more clearly measured from color bar and the standard deviation values in inset of each sample mapping. Moreover, peak wavelength mapping of the V-pit sample shows longer wavelength in almost all areas compared to the standard sample, which is also consistent with the macroscopic PL result. Based on these results, we believe that deep spatial potential fluctuations exist in the V-pit sample because of strain relaxation caused by the large sized V-pits. Although large-scale localization potential is observed from NSOM PL mapping, small-scale localization potential is difficult to be observed by NSOM PL due to the limited spatial resolution of NSOM. Small-scale localization potential strongly affects the carrier transport and recombination compared to large-scale localization potential. Therefore, small-scale localization potential should be considered for estimation of potential fluctuations in each sample. Even though it is difficult to be observed directly from NSOM PL mapping image, it can be indirectly estimated from the broadening of the near-field spectra of each sample^39^,^42^,^45^. Average FWHM values of the standard and V-pit sample at 100 μW are 13.8 nm (83 meV) and 23.3 nm (133 meV), respectively. Because the total broadening is expressed by the sum of the homogeneous and inhomogeneous components, homogeneous broadening should be considered before comparing two average FWHM values of each sample. We note that homogeneous broadening from the semipolar plane of V-pit was not considered in the calculation since the semipolar plane of each sample occupies small portion of the surface. By considering the reported homogeneous broadening value in c-plane InGaN QWs at room temperature (~29 meV)^55^, inhomogeneous broadening values of the standard and V-pit samples are ~54 meV and ~104 meV, respectively. From this result, we can estimate that the V-pit sample has larger inhomogeneous broadening compared to the standard sample due to small-scale localization potential.

**Figure 4:**
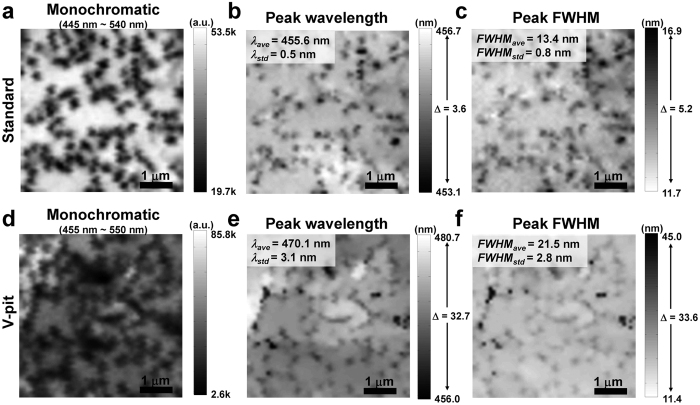
NSOM PL mapping images measured from standard and V-pit samples at 1140 μW laser power. Monochromatic NSOM PL images of the standard and V-pit samples, only obtained from main MQWs wavelength regime, are shown in (**a**) and (**d**). (**b,c**) PL peak wavelength and FWHM mapping images of the standard sample. (**e,f**) PL peak wavelength and FWHM mapping images of the V-pit sample. Inset values (*λ*_*ave*_*, λ*_*std*_*, FWHM*_*ave*_*, FWHM*_*std*_) show average and standard values of the peak wavelength and FWHM. The values of Δ in the scale bars of the peak wavelength and peak FWHM images indicate the differences between the maximum and minimum wavelengths and FWHM, respectively. In the monochromatic and peak FWHM images, we used white and black in the scale bars of the maximum intensity value, respectively, for better comparison with each other.

The correlations and characteristics of the V-pits and potential fluctuations can be more clearly observed in the mappings and monochromatic images of each sample at 1140 μW laser power, as shown in Fig. 4 (monochromatic images are provided in the Supplementary information, Fig. S1). However, in contrast with the low laser power mappings, the potential fluctuations and V-pits feature in high laser power mappings are faded. This fading tendency was also verified in the NSOM PL mappings at various laser excitation powers (Supplementary information, Fig. S2). It can be understood that the ‘emission region’ at high laser power is larger than ‘emission region’ at low laser power because carriers can be more widely diffused when the local carrier density is increased, by considering the ‘emission region’ schematic of I-mode NSOM PL shown in Fig. 2b. However, despite these results, it is difficult to quantitatively describe the influence of V-pits and potential fluctuations using such mappings, because they have much greater spatial information compared to a conventional PL in the same area.

**Figure 5:**
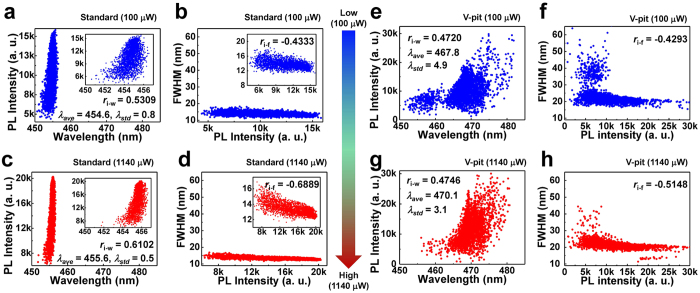
Correlation results of NSOM PL for standard and V-pit samples at 100 μW and 1140 μW laser powers. Correlation results of the PL peak intensity, wavelength and FWHM of the standard sample at (**a,b**) 100 μW, and (**c,d**) 1140 μW laser powers, and those of the V-pit sample at (**e,f**) 100 μW and (**g,h**) 1140 μW laser powers. The scale for all each correlation data was set to be the same. The insets of the standard sample (**a–d**) show the correlation data with the original scale. The correlation coefficient *r*, average peak wavelength *λ*_*ave*_ and standard deviation of wavelength *λ*_*std*_ are also given.

In order to analyze the NSOM PL results, we employed a correlation analysis known as Pearson’s correlation and analyze three parameters: the peak intensity, peak wavelength and FWHM. The correlation analysis provides a quantitative description of the relationship between two parameters. Furthermore, to verify the effect of the V-pits in terms of local carrier density, the results of the NSOM PL at 100 μW and 1140 μW were analyzed and compared. The correlation results of the standard sample at each laser power are given in Fig. 5a,b and c,d, respectively. Figure 5e,f and g,h show the correlation results of the V-pit sample at each laser power, respectively. Each correlation coefficient (*r*) is given in the inset of Fig. 5. In the correlation results, the correlation coefficient between peak intensity and wavelength (*r*_i-w_) of each sample is positive, indicating that the stronger intensity regions have a longer wavelength. On the other hand, the correlation coefficient between the peak intensity and FWHM (*r*_i-f_) of each sample is negative, reflecting that the stronger intensity regions have a smaller FWHM which is in good agreement with the results of Fig. 3. Although these tendencies were previously predicted by comparing each mapping image, the correlation coefficients describe these tendencies more quantitatively. At low laser power, the absolute values of the correlation coefficients (|*r*_i-w_*|,* |*r*_i-f_*|*) of the standard sample (0.5309, 0.4333) are larger compared to those of the V-pit sample (0.4720, 0.4293). At high laser power, |*r|* values of the standard sample (0.6102, 0.6889) show a strong correlation compared to those of the V-pit sample (0.4746, 0.5148). Both results indicate that the standard sample exhibits a strong correlation compared to the V-pit sample. As explained in the experimental section, the size of ‘emission region’ in I-mode NSOM PL is determined by carrier diffusion, which is associated with potential fluctuations (including large-scale and small-scale localization potential) and energy barriers in InGaN/GaN MQWs. Because the NSOM PL mapping images are reconstructed point by point from the ‘emission region’, the point information of the mapping includes the ensemble average information from the whole ‘emission region’. So the sample with shallower (deeper) potential fluctuations/energy barriers would show larger (smaller) size of ‘emission region’ due to longer (shorter) carrier diffusion length. This indicates that the correlation between points of NSOM PL mapping would be stronger (weaker) for the sample with shallower (deeper) potential fluctuations/energy barriers and/or for the stronger (weaker) excitation condition because each point in NSOM PL comes from ensemble average of larger (smaller) size of ‘emission region’ in I-mode NSOM. Therefore, smaller |*r|* values of the V-pit sample compared to those of the standard sample reflect that V-pit sample has smaller size of ‘emission region’ due to deeper potential fluctuations and energy barriers. We note that these potential fluctuations are associated with the small-scale localization potential results obtained from inhomogeneous broadening of near-field spectra in each sample.

To further study the influence of V-pits and potential fluctuations in each sample, the changes in correlation coefficients (Δ*r*) were investigated with varying laser excitation power. We observed that the absolute values of Δ*r* (|Δ*r*_i-w_*|,* |Δ*r*_i-f_*|*) were (0.0793, 0.2556) and (0.0026, 0.0855) for standard and V-pit samples, respectively. Moreover, it was found that |Δ*r*| values of both samples increased, which means that the correlation became stronger. Similar trend was observed at other laser excitation powers (Supplementary information, Fig. S3a and 3b). It can be understood in terms of ‘emission region’ in I-mode NSOM PL. Because carrier diffusion length is increasing with carrier density, the |Δ*r*| values in both samples can increase due to increasing size of ‘emission region’ with power. However, |Δ*r*| values of the V-pit sample are smaller than those of the standard sample. This can be interpreted that the size of ‘emission region’ of V-pit sample cannot rapidly increase compared to that of standard sample due to suppressed carrier diffusion affected by deeper potential fluctuations and energy barriers. In addition, even though peak wavelength shows a blue-shift tendency in the usual power dependent (far-field) PL experiment because of screening and band filling effects, the average peak wavelength (*λ*_ave_) of both samples exhibits a red-shift tendency and their variation (*λ*_std_) decreases in NSOM PL data, as shown in the inset of Fig. 5a,c and e,g. This is because carriers can more easily diffuse to different deep localization sites surrounded by potential barriers with increasing carrier density, which was confirmed at various laser excitation powers (Supplementary information, Fig. S3c and d). Especially, the red-shift of wavelength in the V-pit sample is larger compared to that in the standard sample. By considering this in connection with carrier diffusion, we can also suggest that the V-pit sample has deeper localization potential compared to the standard sample.

**Figure 6:**
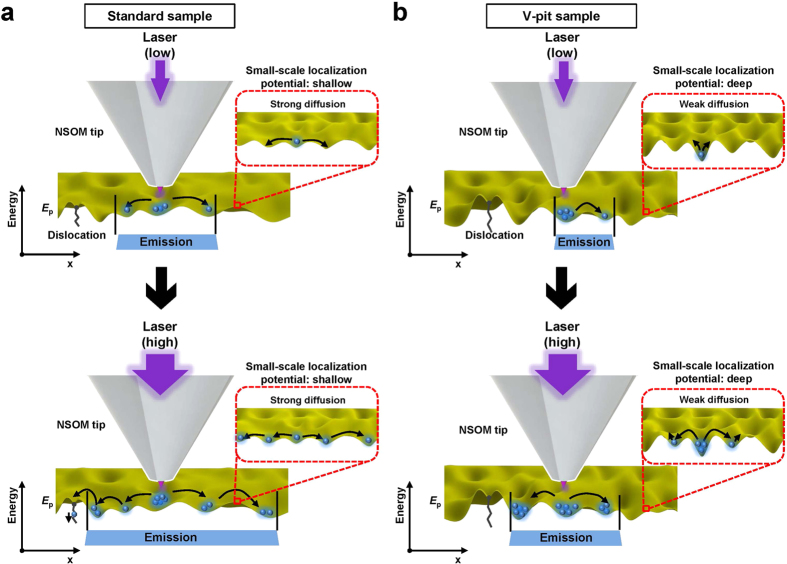
Schematics of possible carrier transfers in the standard and V-pit samples at low and high laser powers. From the results of NSOM PL mappings and correlation analysis, possible carrier transfer behaviors in the potential fluctuation *E*_p_ of the standard and V-pit samples at low and high laser powers are shown in (**a**) and (**b**), respectively. Because of the shallow potential fluctuations including large-scale and small-scale localization potential or low energy barriers of the standard sample, the size of emission region of the standard sample is larger than that of the V-pit sample. Furthermore, it becomes broader with increasing laser power because carriers easily escape from the local potential fluctuations for the same reason. In contrast, the size of ‘emission region’ of the V-pit sample is smaller than that of the standard sample because carriers cannot widely diffuse, due to the deep potential fluctuations or high energy barriers. These carrier transfer is represented as correlation coefficients from I-mode NSOM PL and correlation analysis.

We emphasize that the concept of ‘emission region’ in I-mode NSOM PL combined with the correlation analysis can be successfully applied to investigate the influence of the V-pits and potential fluctuations on local optical properties of InGaN/GaN QWs. With reference to the results previously reported in semipolar^43^ and nonpolar^42^ InGaN QWs using illumination-collection mode NSOM PL system, we can interpret that our results describe quite reasonably the carrier transfer behavior in c-plane, polar InGaN QWs samples with different V pits. For instance, the increment of *r*_i-w_ and the red-shift tendency of λ_ave_ can be attributed to carrier redistribution caused by carrier diffusion in high and low localization potential. In addition, the negative increment of *r*_i-f_ with power in correlation results can be interpreted as the diminished contribution of localized potentials with increasing photoexcited carrier density. We also found that |Δ*r*_i-f_| values of the V-pit sample are smaller than those of the standard sample, indicating that carriers in the V-pit sample have deep localization potential which is able to strongly capture carriers. Moreover, we observed that the double peak shape of the V-pit sample at low power is caused by shorter wavelength emission from high-energy, thinner QWs grown at the inclined facet of V-pit. (near-field emission spectra of V-pit containing and V-pit free regions are given in supplementary, Fig. S4). At high power, however, the double peak shape of the V-pit sample disappears due to the increased size of ‘emission region’ with power. The correlation results suggest that the size of ‘emission region’ of the V-pit sample is always smaller than that of the standard sample because carrier transport in the V-pit sample is suppressed due to the influence of the high energy barriers and the deep potential fluctuations (small-scale localization potential fluctuation), as shown in Fig. 6. Therefore, we can conclude that the V-pit sample has deep localization potential fluctuations and high energy barriers.

In summary, the influence of V-pits and potential fluctuations on local optical properties in InGaN/GaN MQWs was investigated by a nanoscopic PL measurement using NSOM PL with I-mode. To observe the effect of the V-pits and potential fluctuations, the standard and V-pit samples with different sized V-pits were used and compared. From the NSOM PL mapping results, the spatially resolved luminescence features including potential fluctuations were observed. By comparing two samples using power dependent NSOM PL with I-mode and correlation analysis, we not only clearly observed luminescence features but also quantitatively confirmed that the effects of the V-pits and potential fluctuations depend on the V-pits size based on the concept of ‘emission region’ in NSOM PL with I-mode. As a result, it was confirmed that the V-pit sample had high energy barriers and deep localization potential fluctuations. These results suggest that the high energy barriers and deep localization potential fluctuations, which are affected by the size of the V-pits, could be considered one of the reasons for the enhanced optical and electrical performance of the V-pit sample. In conclusion, we successfully observed the spatially resolved luminescence of InGaN/GaN MQWs using nanoscopic measurement. We also quantitatively confirmed the optical effects of the V-pits and potential fluctuations using I-mode NSOM PL and correlation analysis, by comparing two samples with different sized V-pits. Characterizing the optical properties of V-pits and potential fluctuations using the I-mode NSOM PL and correlation analysis methods allowed us to explain the enhancement in LED efficiency provided by MQWs with V-pits.

## Methods

### Experimental set-up

A home-built tuning fork based NSOM was employed for nanoscopic PL measurements, using an aluminum coated optical fiber tip with ~100 nm aperture (MF001, NT-MDT, Russia). A 405 nm diode laser (L405P150, Thorlab, USA) was used as the excitation source to excite the InGaN/GaN MQWs samples. The excitation source was coupled with optical fiber using an optical fiber coupler (9131-M, Newport, USA) and illuminated the NSOM tip through an optical fiber. The luminescence of the sample was collected by an objective lens. Finally, the luminescence of the sample was filtered by a long-pass filter (LP02–407RU-25, Semrock, USA) and spectrally resolved using a monochromator (SP-2300i, Princeton Instruments, USA), and then measured using an electron multiplying charge coupled detector (ProEM, Princeton Instruments, USA). For the power dependent NSOM PL measurement, each level of laser power was measured in front of the optical fiber coupler. The results of the NSOM PL were analyzed using home-built software.

## Additional Information

**How to cite this article**: Kim, M. K. *et al*. Investigating carrier localization and transfer in InGaN/GaN quantum wells with V-pits using near-field scanning optical microscopy and correlation analysis. *Sci. Rep.* **7**, 42221; doi: 10.1038/srep42221 (2017).

**Publisher's note:** Springer Nature remains neutral with regard to jurisdictional claims in published maps and institutional affiliations.

## Supplementary Material

Supplementary Information
