# Supplementary material for: Investigating carrier localization and transfer in InGaN/GaN quantum wells with V-pits using near-field scanning optical microscopy and correlation analysis
Source: Sci Rep. 2017 Feb 13;7:42221. doi: 10.1038/srep42221 (PMC5304185; doi:10.1038/srep42221)
Supplement: Supplementary Information [file srep42221-s1.pdf]

Investigating carrier localization and transfer in InGaN/GaN quantum wells with V-pits using near-field scanning optical microscopy and correlation analysis

MinKwan Kim[1], Sunghan Choi[2], Joo-Hyung Lee[2], ChungHyun Park[2, 3], Tae-Hoon Chung[4], Jong-Hyeob Baek[4], and Yong-Hoon Cho[2,3*]

[1]Graduate School of Nanoscience and Technology, Korea Advanced Institute of Science and Technology, Daejeon 34141, Republic of Korea.

[2]Department of Physics, Korea Advanced Institute of Science and Technology, Daejeon 34141, Republic of Korea.

[3]KI for the NanoCentury, Korea Advanced Institute of Science and Technology, Daejeon 34141, Republic of Korea.

[4]LED Research and Business Division, Korea Photonics Technology Institute, Gwangju 61007, Republic of Korea.

*Correspondence:

Prof. Yong-Hoon Cho

Department of Physics, Korea Advanced Institute of Science and Technology, Daejeon 34141, Republic of Korea. Tel: (82) 42-350-2549, Email: yhc@kaist.ac.kr

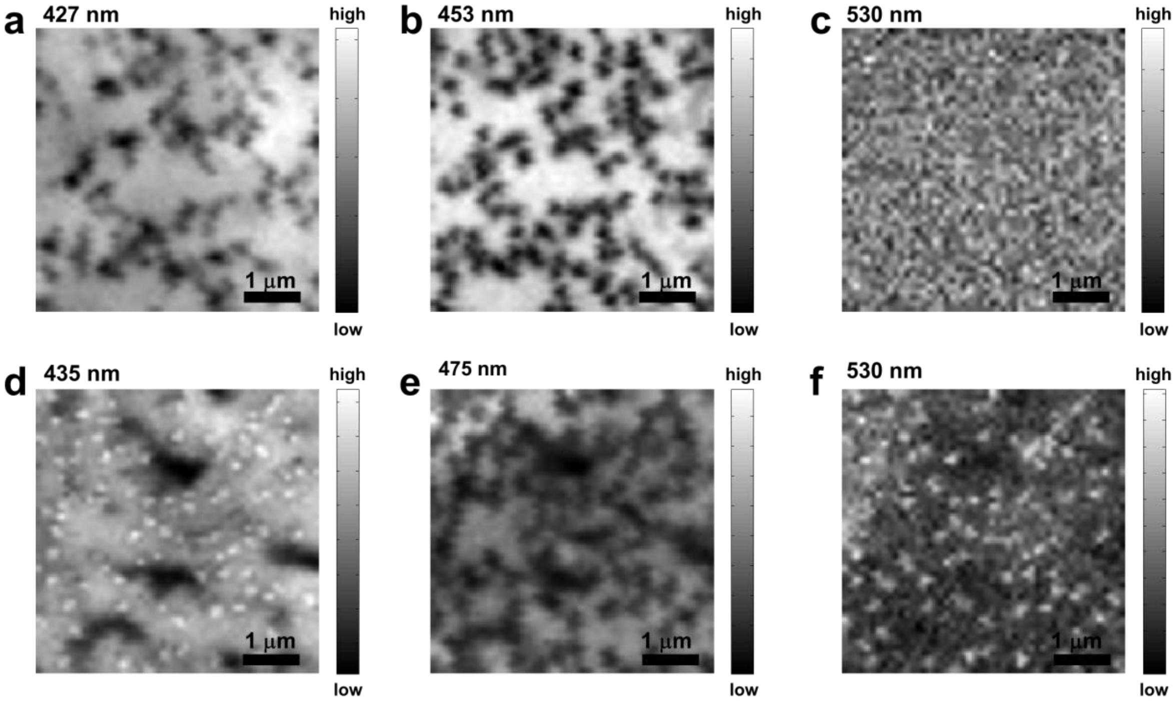

Figure S1| Monochromatic images of NSOM PL obtained from the standard and V-pit samples at 1140 μW laser power. Monochromatic images of the standard and V-pit samples are given in (a-c) and (d-f). In the short wavelength regime, which includes the InGaN/GaN SLs wavelength, the NRC regions of the V-pit sample show a strong intensity tendency, whereas the standard sample clearly shows the NRCs as a low intensity region. It is thought that emission of InGaN/GaN SLs scattered by large size V-pits in the V-pit sample makes the NRC regions, which originally has low intensity in monochromatic image, but appears to have strong intensity. Also, while both samples show clearly the NRC area in the main MQWs wavelength regime, bright spots are only observed in the V-pit sample in the long wavelength regime. It is thought that indium segregation exists at the center of the large sized V-pits. These V-pit effects, such as scattering and indium segregation, are well known characteristics of V-pits.

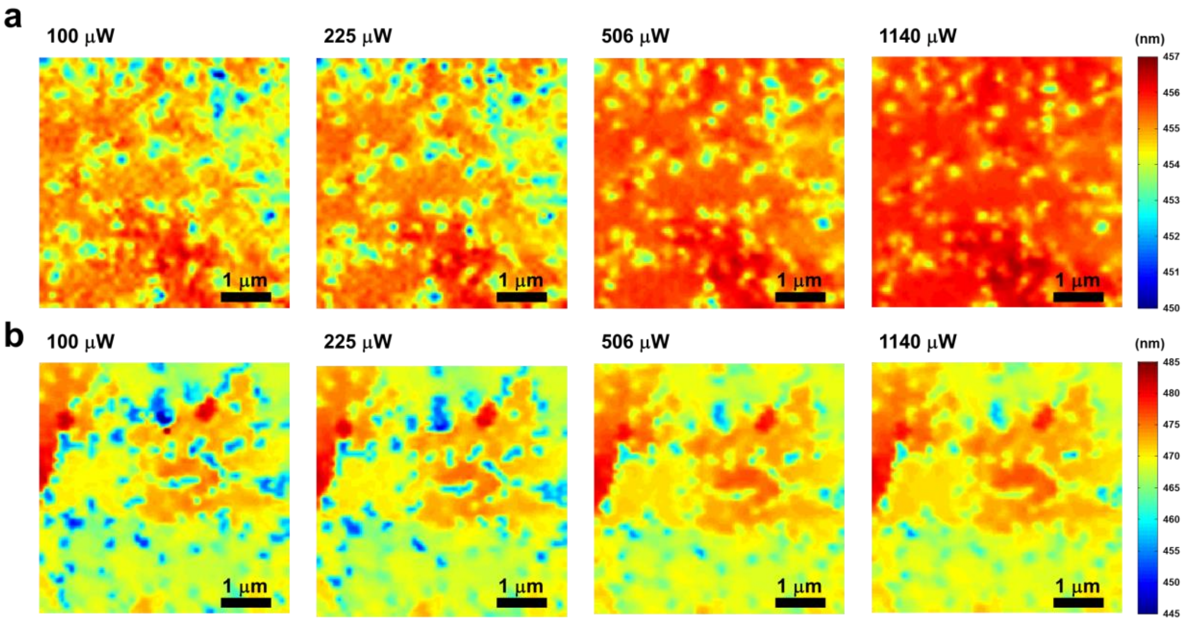

Figure S2| Peak wavelength mapping images of NSOM PL at various laser excitation powers. (a) Peak wavelength NSOM PL mapping images of the standard sample for several laser powers. The color bars of all the images have been adjusted to the same scale. (b) Peak wavelength NSOM PL images of the V-pit sample for several laser powers. The color bars have also adjusted to the same scale. As shown in the peak wavelength mapping images for each sample, detailed features of the potential fluctuations and V-pits have faded with increasing laser power. Furthermore, red-shift tendency is observed in both samples.

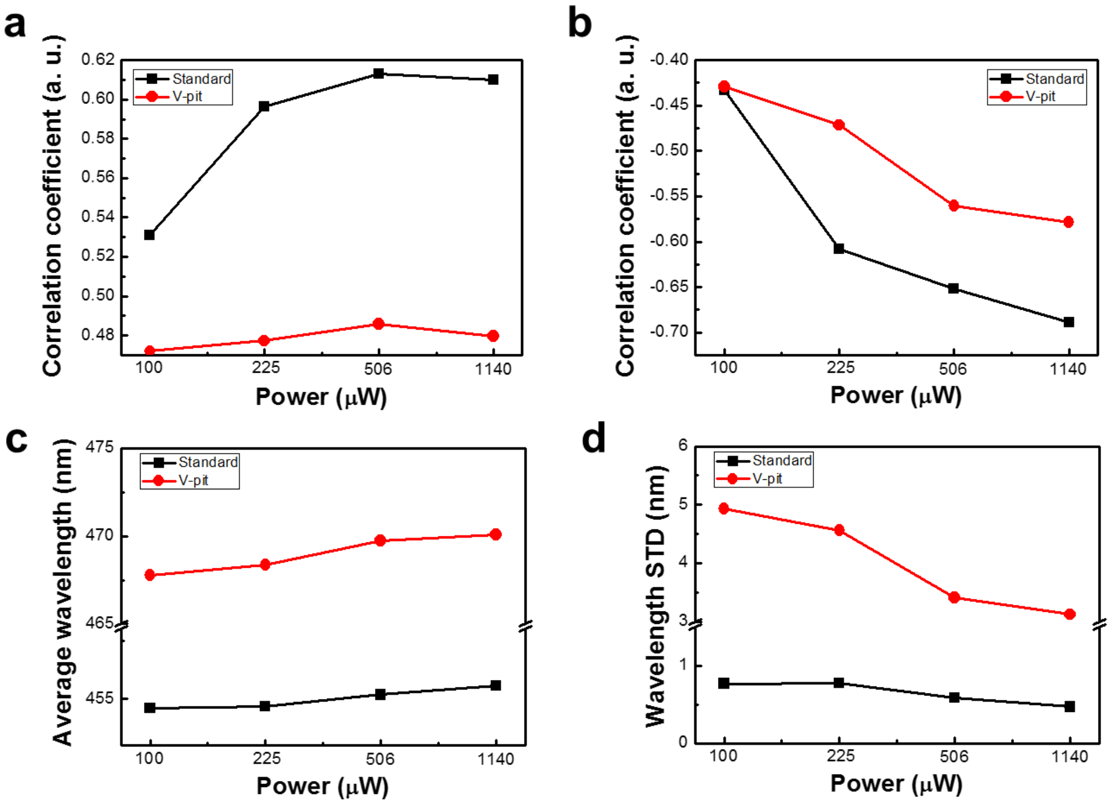

Figure S3| Graphs of correlation coefficient, average peak wavelength and standard deviation of peak wavelength obtained from the standard and V-pit samples at various laser excitation powers. (a) Intensity-wavelength correlation coefficient of each sample at various laser excitation powers. It shows increasing correlation tendency with increasing laser power despite of a little reduction at high power regime. (b) Intensity-FWHM correlation coefficient of each sample at various laser excitation powers. Absolute value of it also shows increasing correlation tendency with increasing laser power. (c) Average peak wavelength of each sample at various laser power. Both samples show red-shift tendency with increasing laser power. (d) Standard deviation of wavelength of each sample at various power. Both sample show decreasing tendency with increasing laser power.

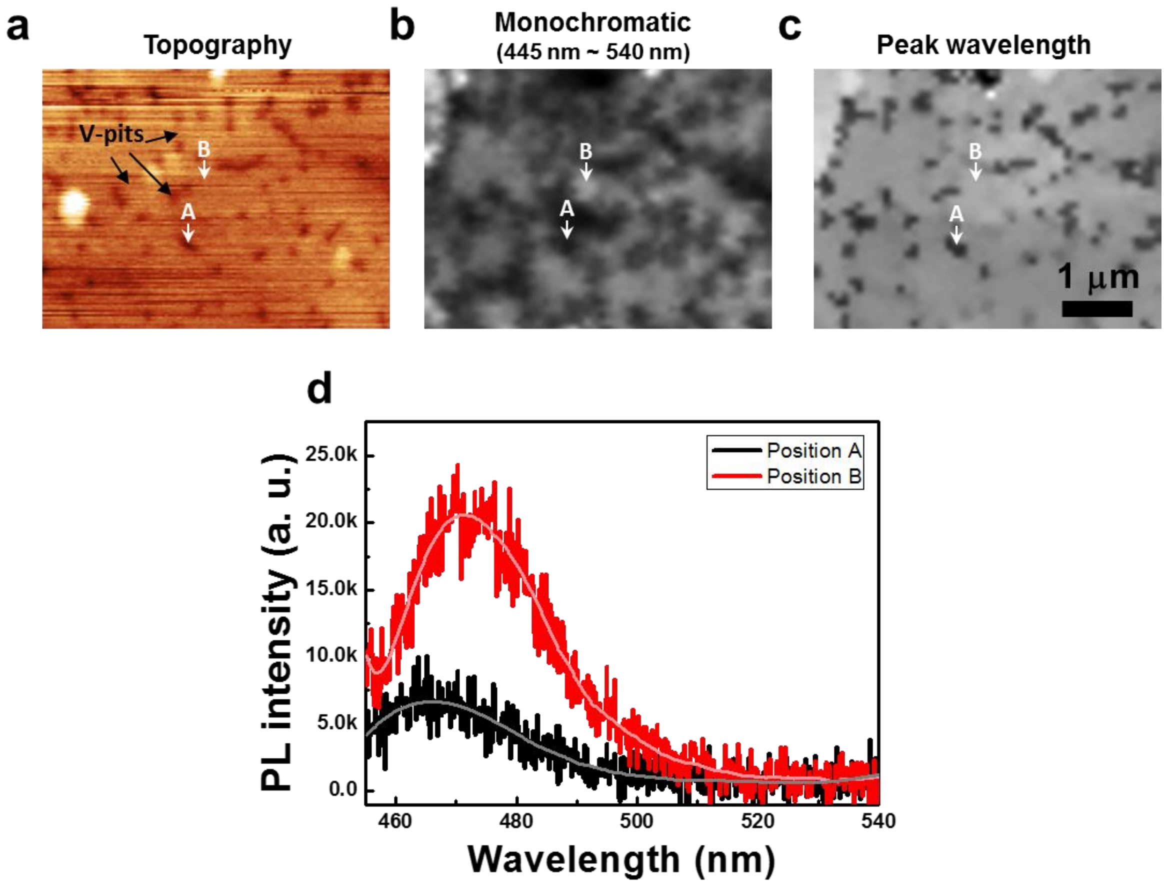

Figure S4| Topography image with monochromatic and peak wavelength images of the V-pit sample at 100 μW and the near-field spectra of V-pit containing and V-pit free regions. (a) Topography image obtained from NSOM PL. (b) Monochromatic NSOM PL image of the V-pit sample at 100 μW. (c) Peak wavelength image of the V-pit sample at 100 μW. (d) The near-field spectra of V-pit contacting region (position A) and V-pit free region (position B). These near-field spectra only show main MQWs wavelength range without InGaN/GaN SLs.
